# A Case of Solitary Fibrous Tumour Arising From the Anterior Skull Base

**DOI:** 10.7759/cureus.19733

**Published:** 2021-11-19

**Authors:** Rahul Pankhania, Yu Lau, Gentle Wong, San Sunkaraneni

**Affiliations:** 1 Ear, Nose & Throat, Royal Surrey County Hospital, Guildford, GBR

**Keywords:** otolaryngology, ent, anterior skull base, rhinology, rhinorrhoea, nasal obstruction, solitary fibrous tumour

## Abstract

Solitary fibrous tumours (SFTs) are rare tumours of mesenchymal origin, most commonly found in adults with a median age of 45-50 years. Cases in the head and neck are uncommon, and definitive diagnosis relies on histomorphological features supported by immunohistochemical staining. Here we present a case of a 26-year-old gentleman with a one-year history of right-sided nasal obstruction and bloody discharge on a longstanding background of recreational cocaine use. A computed tomography scan of the paranasal sinuses demonstrated a mass arising from the anterior skull base extending into the right middle turbinate. Immunohistochemistry testing for tumour characterisation showed hematopoietic progenitor cell antigen (CD34) and signal transducer and activator of transcription 6 (STAT6) positivity in keeping with an SFT. The patient underwent definitive surgery via endoscopic piecemeal resection with no further reoccurrence at follow-up.

## Introduction

Solitary fibrous tumours (SFTs) are rare tumours of mesenchymal origin, first described as primary spindle cell neoplasms of the pleura in 1931 by Klemperer and Rabin [1]. They are commonly found in the pleura with an incidence of 2.8 per 100,000 but are known to present more infrequently in other locations such as the mediastinum, urogenital system and abdomen [1,2]. Cases in the head and neck are uncommon. The tumour initially presents asymptomatically as a soft tissue mass allowing progressive growth, following which symptoms such as epistaxis, nasal obstruction, rhinorrhoea, hyposmia, and facial pain may develop [3]. Herein we present a protracted presentation of an SFT and explore the critical points of diagnosis, investigations and treatment.

## Case presentation

**Figure 1:**
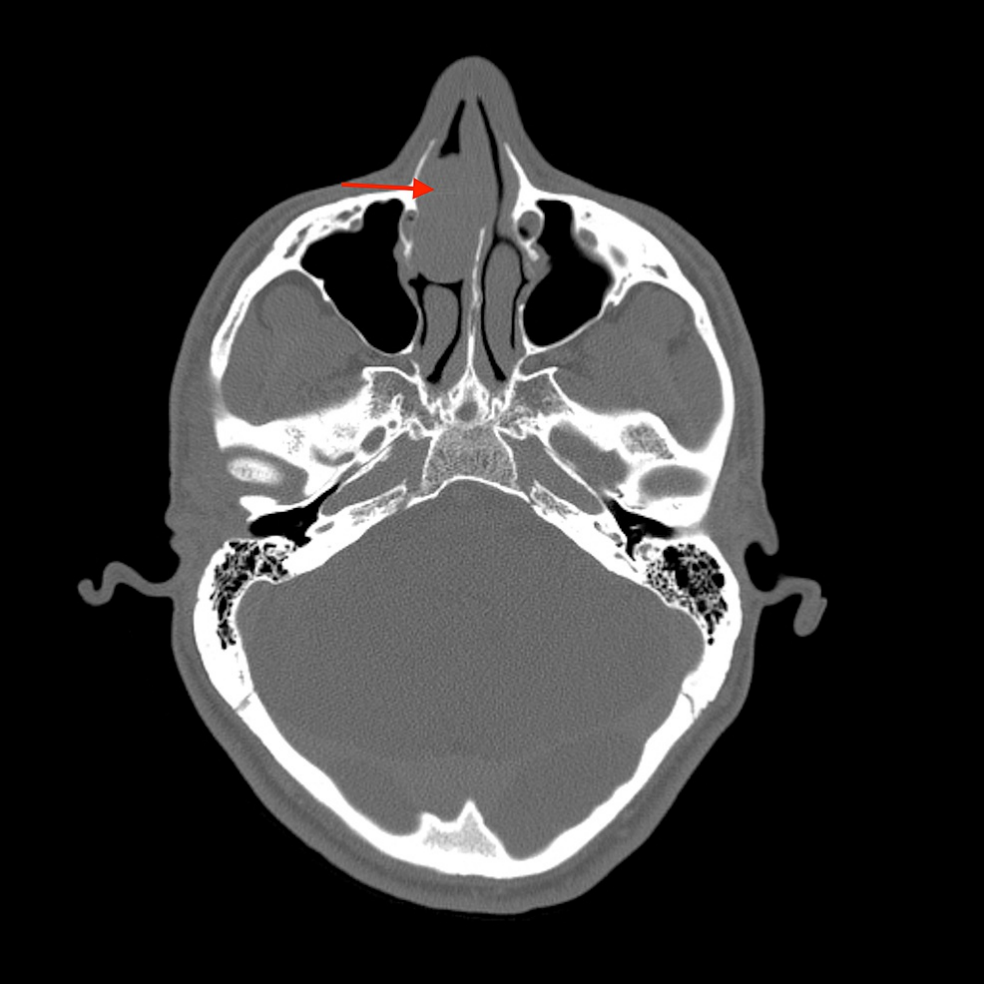
Axial soft tissue contrast-enhanced computed tomography scan of the paranasal sinuses and orbit

**Figure 2:**
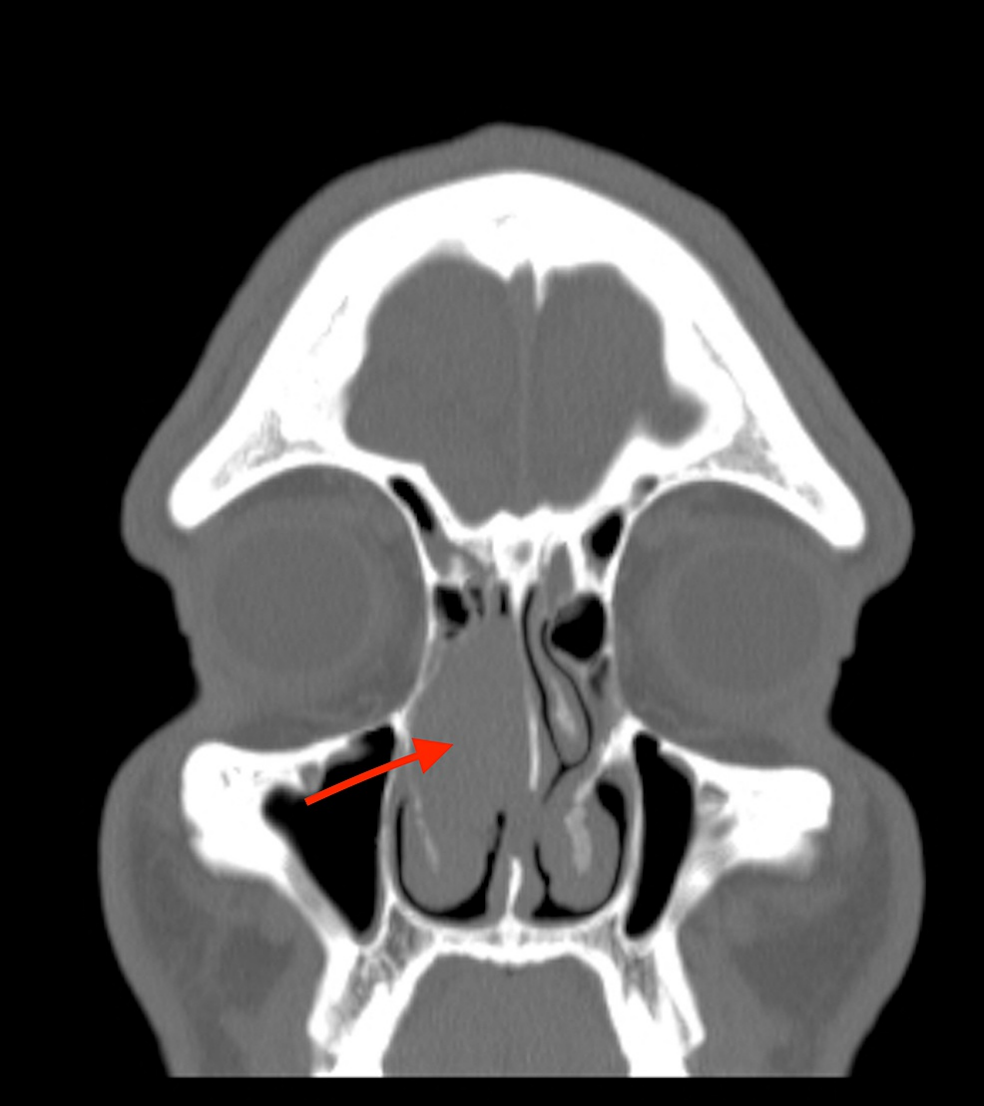
Coronal soft tissue contrast-enhanced computed tomography scan of the paranasal sinuses and orbit

A 26-year old gentleman presented with a one-year history of right-sided nasal obstruction and bloody discharge. There were no sinonasal or associated respiratory symptoms. Anterior rhinoscopy revealed a large fleshy mass present within the right nasal cavity. The contralateral nasal cavity was clear with no associated cervical lymphadenopathy. Flexible nasal endoscopy was not feasible past the nasal vestibule due to the obstruction present. The patient had a longstanding history of recreational cocaine use with no other past medical history. A computed tomography (CT) scan of the paranasal sinuses was undertaken and demonstrated a mass extending into the right middle turbinate from the anterior skull base. The mass displaced the ipsilateral uncinate process and nasal septum to the right and left respectively as seen in Figures 1, 2.

There was no local bony erosion or intra-orbital involvement and intracranial appearances were normal. Differentials to consider based on the clinical presentation and radiological findings included nasopharyngeal carcinoma, haemangiopericytoma and fibrosarcoma.

Diagnostic biopsies (1.0 x 1.0 x 0.3 cm) were performed under general anaesthesia that determined cellular uniform spindle cells infiltrating the nasal mucosa with focal ulceration in keeping with an SFT. Table 1 summarises our immunohistochemistry testing for tumour characterisation.

**Table 1:** Immunohistochemistry testing for tumour characterisation CD34: hematopoietic progenitor cell antigen; STAT6: signal transducer and activator of transcription 6; S-100: Schwannian-100; MUC4: mucin 4, cell surface associated protein; Sox10: SRY-box transcription factor 10; GFAP: glial fibrillary acidic protein; EMA: epithelial membrane antigen

Immunohistochemistry test	Result
CD34	Positive
STAT6	Positive
Desmin	Negative
S-100	Negative
MUC4	Negative
Sox10	Negative
GFAP	Negative
EMA	Negative

The patient underwent definitive surgery via endoscopic piecemeal resection where the vascular tumour was found to arise near the first olfactory fibre. Intraoperative pathology samples revealed three separate tumour areas. Post-operatively, the patient was observed with no complications and subsequently discharged the same day with a course of oral steroids and nasal sinus washouts. At the 15-month follow-up, the patient had no further nasal obstruction and continued to remain symptom free with no reoccurrence.

## Discussion

SFTs were first described by Wagner in 1879 and further characterised by Klemperer and Rabin in 1931 [1]. Most have mesenchymal origin and arise from serous membranes such as pericardium, peritoneum, and pleura, but soft-tissue SFTs also occur and these can appear in the orbit, thyroid, nasopharynx, larynx, and salivary glands [1,4]. To the best of our knowledge, our report is only the second in SFTs reported involving recreational drug use and adds to the paucity of cases in the head and neck region.

Most SFTs occur in adults with a median age of 45-50 years and a mean age of of 50.4 years, in equal distribution between men and women [5-6]. Extrathoracic SFTs generally present with symptoms attributable to a mass or pressure effect depending on location [7]. In the nasal cavity, the commonest presentation is nasal obstruction; other symptoms include epistaxis, rhinorrhoea, hyposmia, and epiphora [5]. Radiological findings are often non-specific with SFTs described as having well-defined lobulated contours with discrete margins. [8]. However, a review of SFTs' radiological appearances identified that due to the highly vascular nature, the presence of a large collateral feeding vessel or visible fatty component can be a helpful feature to aid clinicians and radiologists in narrowing the differential diagnosis adding further importance to preoperative imaging [8].

Definitive diagnosis relies on histomorphological features supported by immunohistochemical staining and increasingly, molecular analysis to distinguish SFTs from other tumours. Microscopically, SFTs are hypocellular collagen-rich areas mixed with a proliferation of uniform spindle cells. Almost all SFTs express CD34 positivity, which is a myeloid progenitor cell antigen also present in endothelial cells. Signal transducer and activator of transcription 6 (STAT6) has recently been identified as a consistent finding in SFTs [9,10]. Vimentin is commonly positive, but this is non-specific and expressed by most mesenchymal and many epithelial neoplasms. SFTs do not show immunoreactivity for keratin, desmin, S-100 protein and glial fibrillary acidic protein. We also tested for mucin 4, cell surface associated protein (MUC4) that is a highly sensitive and specific marker for low-grade fibromyxoid sarcoma [10]. SRY-box transcription factor 10 (Sox10) is a marker not only for schwannian and melanocytic tumours of soft tissue and skin, but also myoepithelial or basal cell tumours [9]. Molecular analysis has increasingly found gene fusion of the transcriptional repressor, nuclear polyadenosine RNA-binding 2 (NAB2) with the transcriptional activator STAT6, forming NAB2-STAT6, as the defining driver mutation of SFTs [11].

Surgical removal is curative and the definitive management option, with resectability identified as the single most important indicator of clinical outcomes [12]. However, tumours measuring more than 10 cm in diameter are associated with a worse prognosis due to a high chance of reoccurrence and metastatic spread that requires close follow-up post-surgery [9]. Demicco et al. devised a risk stratification system to evaluate the risk of reoccurrence and metastasis for SFTs as seen in Table 2. This is a useful scale that may allow clinicians to determine the degree of follow-up patients may require and plan for further treatment options [13].

**Table 2:** Solitary fibrous tumour risk stratification system

Risk factor	Score
Age	
<55	0
≥55	1
Tumour size	
<5	0
5 to <10	1
10-15	2
≥15	3
Mitotic count (per 10 high-power fields)	
0	0
1-3	1
≥4	2
Tumour necrosis	
<5%	0
≥10%	1
Risk class	Total score
Low	0-3
Intermediate	4-5
High	6-7

The effects of cocaine as a vasoconstrictor and an irritant to the epithelium on the nasal mucosa are well cited in the literature with the recurrent use resulting in mucoperichondrial ischaemia, inflammation, and localised necrosis of the tissues often leading to septal perforation [14]. In our case, the patient had a longstanding history of regular cocaine use. Whilst the exact causal relationship between cocaine use and tumour formation is unknown, we postulate that due to repeated vasodilation and vasoconstriction, primitive fibroblastic cells involved in tissue remodelling may acquire neoplastic growth potential leading to the formation of neoplasms such as SFT [15-16].

## Conclusions

Solitary fibrous tumour is a rare and unusual pathology particularly in the nasal and paranasal region. It should be considered as a differential diagnosis in patients presenting with nasal obstruction and bloody rhinorrhoea and the use of cocaine may be a predisposing factor. Diagnosis relies on histomorphological features supported by immunohistochemical staining. Surgical intervention is the definitive management; however, there is a high risk of intraoperative bleeding due to the vascular nature of the tumour. Residual tumours may benefit from post-operative stereotactic radiation.
